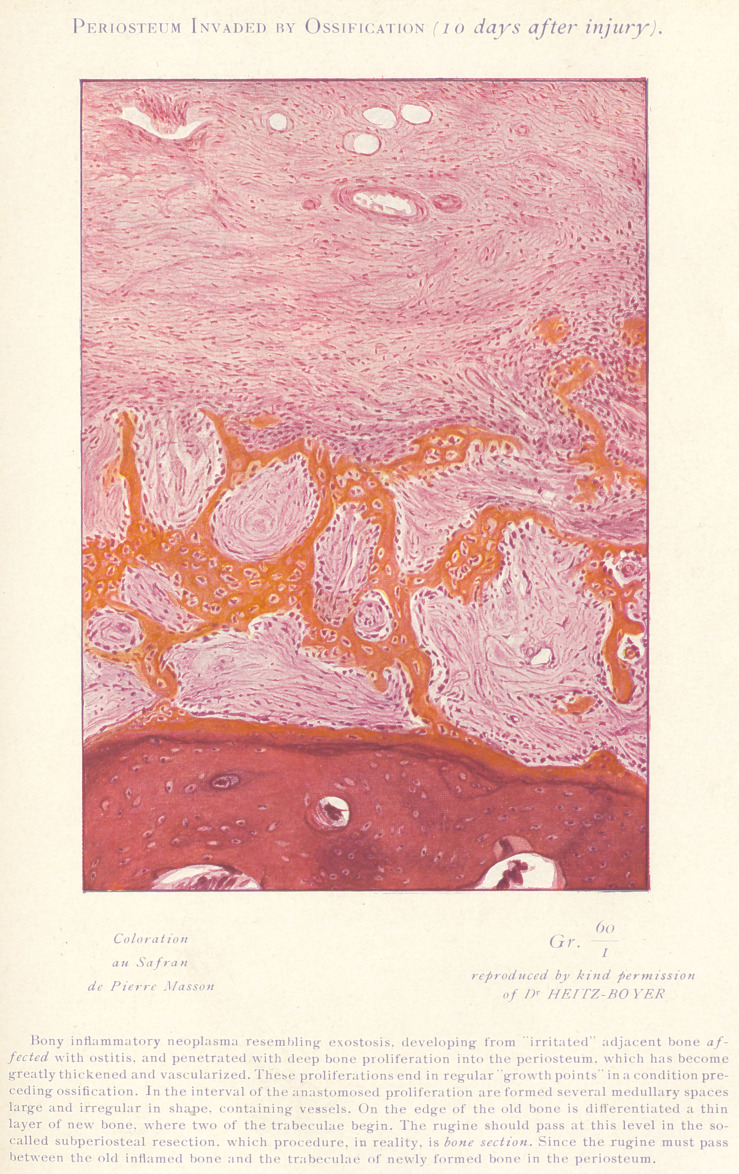# Treatment of Fractures of the Femur

**Published:** 1918-09

**Authors:** 


					﻿Treatment of Fractures of the Femur. By Medecin-Major
Leriche.
The speaker remarked that the gravity of a fracture differs accord-
ing to the distance from the front at which the inspection is made.
In a general way the nearer to the line, the more serious the frac-
tures seem to be.
At an ambulance 6 kilometers from the line in 1917, while things
were quiet, fractures of the femur gave a mortality of 66 0/0.
6 kilometers further back the mortality was only 32 0/0, and lately
in a spot 100 kilometers from the line the mortality was nil.
The speaker referred merely to fractures observed during the first
hours.
It was believed at first that indications for primary amputations
in fractures of the thigh had become very rare; but the figures just
mentioned prove that it is not so.
At the present time it is considered that immediate amputation
ought to be performed under the following circumstances :
When a shocked patient
has not come out of his
shock within two hours after
proper treatment.
When there is a lesion of
the vessels of thigh, femoral
artery, or popliteal vessels.
When the patient has been
wearing a tourniquet for sev-
eral hours. When a patient
with a fracture of the thigh
has had one on for three or
four hours, the conservative
operation becomes very se-
rious and as a rule such cases
die of septicemia in twenty-
four hours.
When there are, several
wounds all of which need
urgent treatment. In those
cases amputation is very ne-
cessary for it shortens the
intervention.
Fortunately the majority
of fractures are not so se-
rious, and recovery can be
obtained by conservative
treatment. In cases without
shock spinal anesthesia is
employed. For shocked ca-
ses the speaker uses ethyl chloride or ether in small doses.
In the intervention itself, there are three points to be consid-
ered :
(i) Treatment of soft parts — this must be carried out on the
same lines as for ordinary wounds of the soft parts; with expision
of all torn tissues, and progressive immediate hemostasis as
the bone is approached; otherwise a great number of veins
which bled at the time of the operation stop bleeding at the end of
it, a condition which might cause secondary hemorrhage. The
incisions vary according to the tract of the projectile. The object
is to have a clean surgical wound. It is only then that the bone can
be treated-*-
(2) As to the treatment of the bone, the author believes that dis-
cussions as to the extent of
esquillectomy are useless.
The object of intervention
is to follow out the tract
and to establish correct ana-
tomical conditions, and the
treatment of the bone must
be judged accordingly.
He considered three
cases :
n) The projectile has not
reached the bone; after the
incision of the soft parts the
projectile is reached and
behind it there is healthy
muscle, therefore it is not
the projectile that has caus-
ed the direct fracture of the
thigh. This first variety is
that of accidental fractures
in wounds of the thigh.
b') The projectile has come
in direct contact with the
bone, has broken it, but has
not penetrated inside. Such
fractures have been called
by military surgeons “ frac-
tures by contact ”. These
cases are not very serious.
The only thing to bear in
mind is that the projectile
might drive in debris of
clothing or foreign bodies which must be sought out. These frac-
tures "by contact must not be confused with the third category
which includes real war fractures.
The projectile has penetrated into the bone and has come out
on the other side. The object of the operation must be the same
as in the treatment of all war wounds. The complete tract of the
projectile must be cleaned so that no foreign body is overlooked.
The bone is widely opened. The medullary, canal is cleaned out
and loose splinters removed. The adherent splinters must not be
removed as long as the tract to the projectile isopen to view. But
such is not the case when the projectile has passed inside the
medullary canal and the splinters have gathered by muscular
contraction. However, enough bone must be taken out to allow
the thorough cleansing of the medullary canal.
One or more splinters of bone are removed in order to open the
medullary canal to view. One might fear, by removing those
splinters, to diminish the value of the osteogenetic reparation, and
by removing one or two splin-
ters to compromise later solid-
ity of the bone and cause
pseudarthrosis.
If the subperiosteal operation
is performed, it must be done
in a special way, with a very
sharp instrument, and on the
deep side of the fibrous perios-
teum a small amount cf bone
dust must be left.
When the operation has been
carried out in this way, osteo-
genesis begins very fast. The
author has studied this matter
very thoroughly. On the fourth
day, bone started to grow around the bope dust and as early as the
ninth day an X-ray picture could be obtained, and on the thirty-
fifth or fortieth day, consolidation was generally obtained. This
kind of intervention makes possible a very large callus, which
grows on one side of the bone and which gradually becomes smal-
ler. But never has the speaker seen pseudarthrosis occur.
Leriche thinks that in cases of fracture, there is no need for
primary suture. He has performed several but has sometimes been
obliged to reopen the wound.
When the operation on the bone and the soft parts is finished, he
puts on an aseptic dressing and immobilizes the limb in extension
and suspension. He renews the dressing three days later under
anesthesia, and, if the wound looks healthy, sutures it. The suture
is performed in several layers. If the case is not very severe, the
suture can be started on the 3rd day, continued on the 5th, then on
the 7th; and the operation is generally completed on the 10th day.
The author thinks that delayed primary suture can be done
under these conditions, in almost all fractures with wound of the
soft parts, and almost all the fractures by contact, except that
delayed primary suture in fractures with through-and-through
wounds is successful in only 30 to 35 0/0 of the cases.
The author communicates the following figures, based on obser-
vations extending over a six months period: 17 cases of fractures
of the femur, out of which there were 4 primary sutures all success-
ful; 6 delayed primary sutures, also all successful; and 7 fractures
with through-and-through wounds, only 2 of which could be sutured
after 10 days. The proportion, it can be seen, varies in the three
classes.
				

## Figures and Tables

**Fig. 1. Fig. 2. f1:**
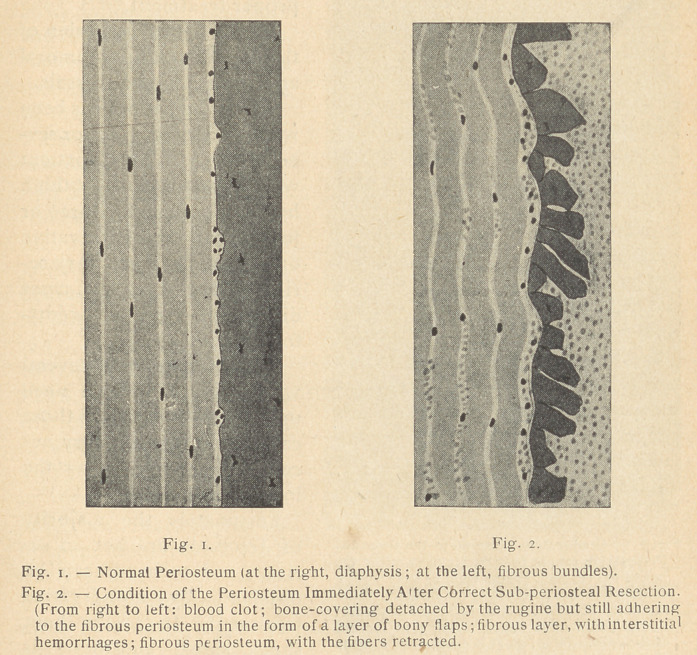


**Fig. 3 f2:**
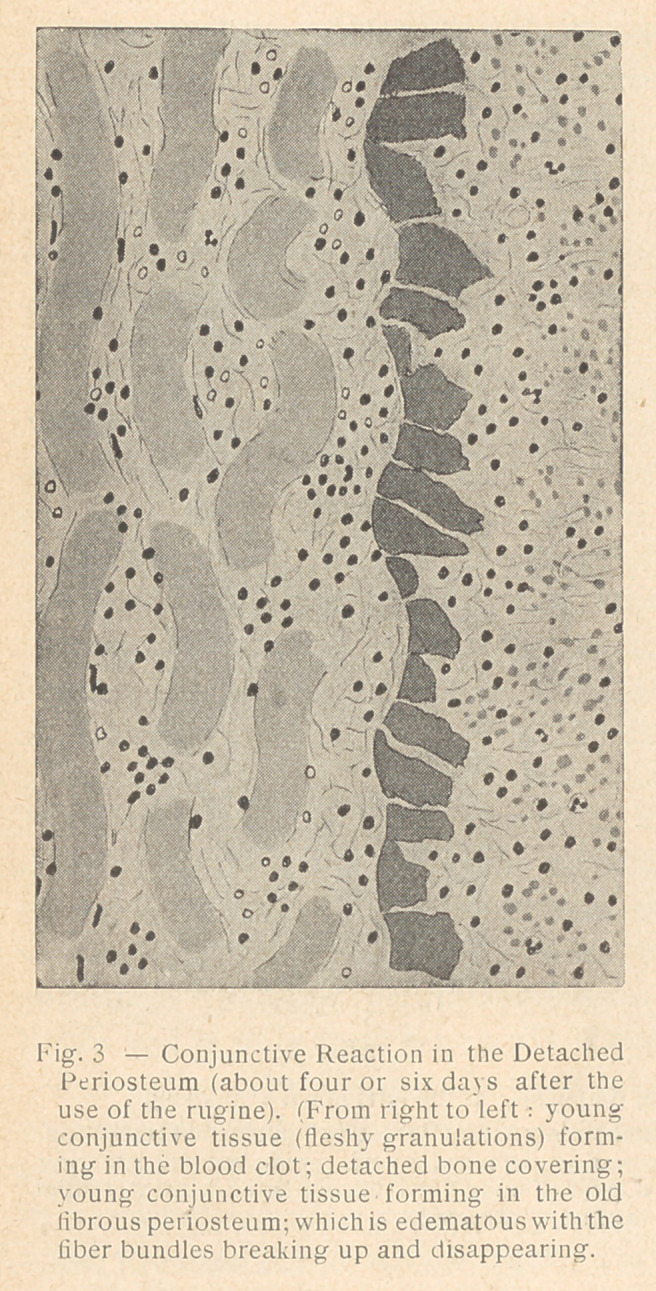


**Fig. 4. f3:**
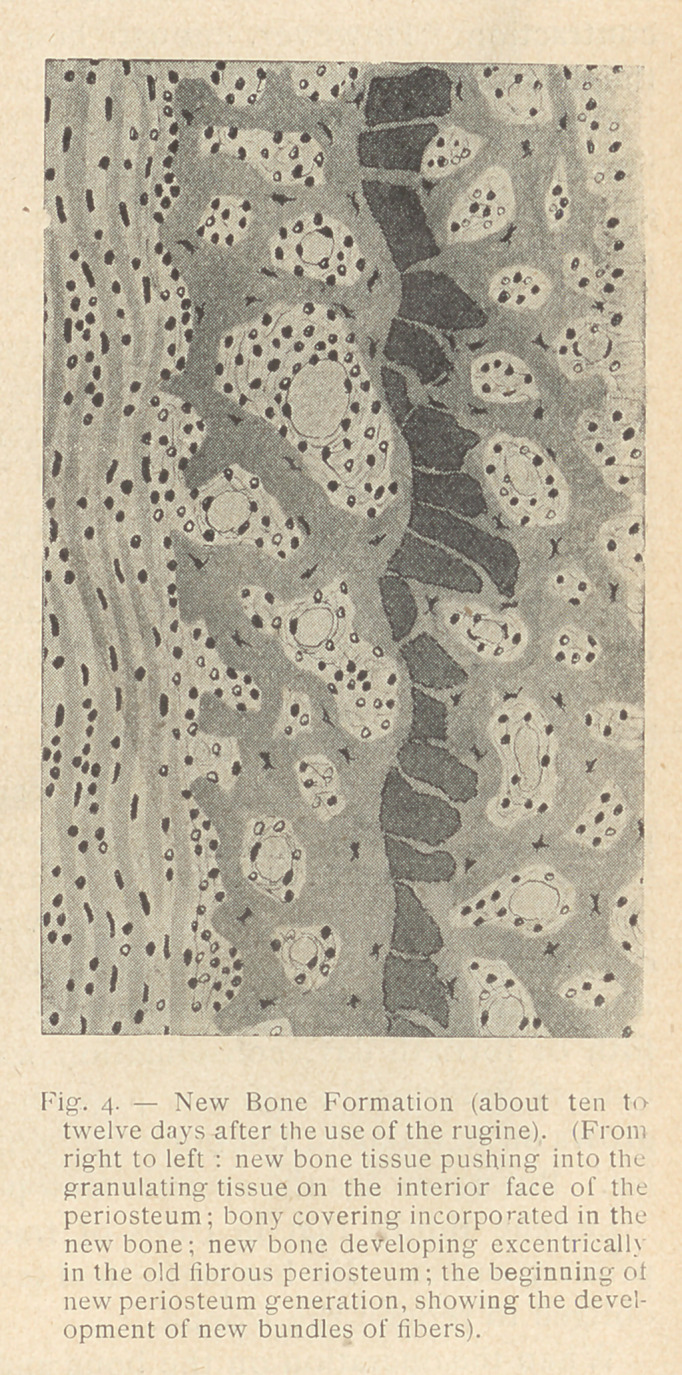


**Fig. 5. f4:**
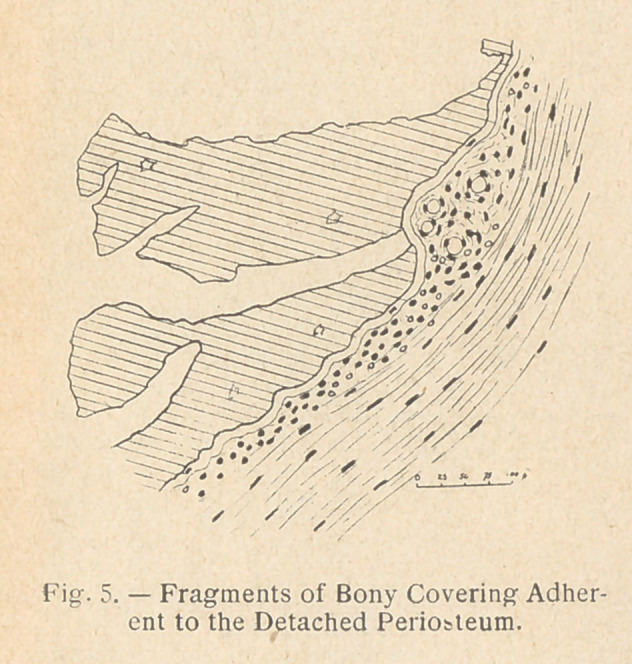


**Figure f5:**